# Atypical ductal hyperplasia on vacuum-assisted breast biopsy: a scoring system to predict the risk of upgrade to malignancy

**DOI:** 10.1007/s11547-023-01740-4

**Published:** 2023-10-24

**Authors:** Rossella Rella, Marco Conti, Alberto Borghetti, Paolo Belli, Francesca Morciano, Claudia Rossati, Andrea Caneva, Alba Di Leone, Gianluca Franceschini, Elisabetta Gori, Francesca Fornasa, Oscar Tommasini, Giovanna Romanucci

**Affiliations:** 1 UOC Diagnostica per Immagini, Ospedale G.B. Grassi, Via Gian Carlo Passeroni, 28, 00122 Rome, Italy; 2 UOC di Radiologia Toracica e Cardiovascolare, Dipartimento di Diagnostica per Immagini, Radioterapia Oncologica ed Ematologia, Fondazione Policlinico Universitario Agostino Gemelli IRCCS, Largo A. Gemelli 8, 00168 Rome, Italy; 3 UOC Malattie Infettive, Fondazione Policlinico Universitario Agostino Gemelli IRCCS, Largo A. Gemelli 8, 00168 Rome, Italy; 4 Facoltà di Medicina e Chirurgia, Università Cattolica Sacro Cuore, Largo F. Vito 1, 00168 Rome, Italy; 5 UOSD Breast Unit ULSS9, Ospedale di Marzana, Piazzale Lambranzi, 1, 37142 Verona, Italy; 6 Divisione di Patologia, ULSS9 Scaligera, Ospedale G. Fracastoro, San Bonifacio, 37047 Verona, Italy; 7 Multidisciplinary Breast Centre, Dipartimento Scienze della Salute della Donna e del Bambino e di Sanità Pubblica, Fondazione Policlinico Universitario A. Gemelli IRCCS, 00168 Rome, Italy

**Keywords:** Atypical ductal hyperplasia, Upgrade, Vacuum-assisted biopsy, Underestimation, Scoring system

## Abstract

**Rationale and objectives:**

Our multicentric study analysed clinical, radiologic and pathologic features in patients with atypical ductal hyperplasia (ADH) diagnosed with vacuum-assisted biopsy (VAB), to identify factors associated with the risk of upgrade, to develop a scoring system to support decision making.

**Materials and methods:**

Patients with ADH on VAB under stereotactic/tomosynthesis guidance (2012–2022) were eligible. Inclusion criteria were availability of surgical histopathological examination of the entire lesion or radiologic follow-up (FUP) ≥ 24 months. VAB results were compared with surgical pathological results or with imaging FUP evolution to assess upgrade. A backward stepwise linear regression was used to identify predictors of upgrade. The discriminatory power of the model was calculated through the area under the receiver operating curve (ROC–AUC); the Hosmer–Lemeshow test was used to assess model calibration. The points system was developed based on the selected risk factors, and the probability of upgrade associated with each point total was determined.

**Results:**

112 ADH lesions were included: 91 (91/112, 81.3%) underwent surgical excision with 20 diagnosis of malignancy, while 21 (21/112, 18.7%) underwent imaging FUP with one interval change (mean FUP time 48 months). Overall upgrade rate was 18.7% (21/112). Age, menopausal status, concurrent breast cancer, BIRADS classification and number of foci of ADH were identified as risk factors for upgrade. Our model showed an AUC = 0.85 (95% CI 0.76–0.94). The points system showed that the risk of upgrade is < 2% when the total score is ≤ 1.

**Conclusion:**

Our scoring system seemed a promising easy-to-use decision support tool for management of ADH, decreasing unnecessary surgeries, reducing patients’ overtreatment and healthcare costs.

## Introduction

Atypical ductal hyperplasia (ADH) is an intraductal clonal epithelial cell proliferation which involves the terminal ductal lobular units (TDLUs). ADH shares with low-grade ductal carcinoma in situ (DCIS) the same histological and architectural features but, when no more than one TDLU is involved and the size of the low-grade intraductal proliferation is less than 2 mm, a diagnosis of ADH is made [1]. ADH usually presents as microcalcifications on mammograms, so its incidence increased after the introduction of population-based screening mammography [2] and it accounts about 15% of pathologic finding of minimally invasive breast biopsies [3, 4].

ADH is classified as lesion “with uncertain malignant potential”, or a “B3" lesion and it is considered a direct but nonobligate precursor as well as an independent risk factor for breast cancer [1]. To date, the last consensus conference on B3 lesions recommends surgical excision of ADH cases (considering follow-up only in special situations after multidisciplinary discussion) [5] due to its risk of upgrade at surgical excision of approximately 25% [6–9]. Therefore, the majority of surgical biopsies for ADH results in benign findings and in a substantially unnecessary surgical procedure. This emphasizes the need to identify women who are more likely to have a cancer and really need surgical excision, avoiding unnecessary surgical breast biopsies with surgical risk for the patient and healthcare costs.

In recent years, there has been an increasing debate over whether selected cases of ADH could receive only follow-up and many studies examined the radiological and histologic characteristics of ADH on percutaneous breast biopsies to determine features that would predict the risk of upgrade at surgical excision [10, 11]. Previous studies and a recent meta-analysis reported that the upgrade rate is lower when stereotactic biopsy is performed, a larger calliper of needle is used and targeted lesion is completely removed [12–15]. However, all data in literature are from single-institution studies.

Our multicentric study analysed clinical, radiologic and pathologic features in a large cohort of patients with ADH diagnosed with VAB under stereotactic/tomosynthesis (DBT) guidance, to identify factors associated with the risk of upgrade to cancer to develop a scoring system to support risk–benefit-based decision making.

## Materials and methods

### Study population

This is a multicentric observational retrospective study. Data were collected at three sites in Italy, and each single-centre study was approved by the local Institutional Review Board (IRB) (Protocol number 0078775/2023, 21/04/2023). Informed consent was obtained from all individual participants included in the study. Data collection and aggregation were performed in a fully anonymized way and in line with international legislation. The study was performed in accordance with the Declaration of Helsinki statement for medical research involving human subjects.

**Fig. 1:**
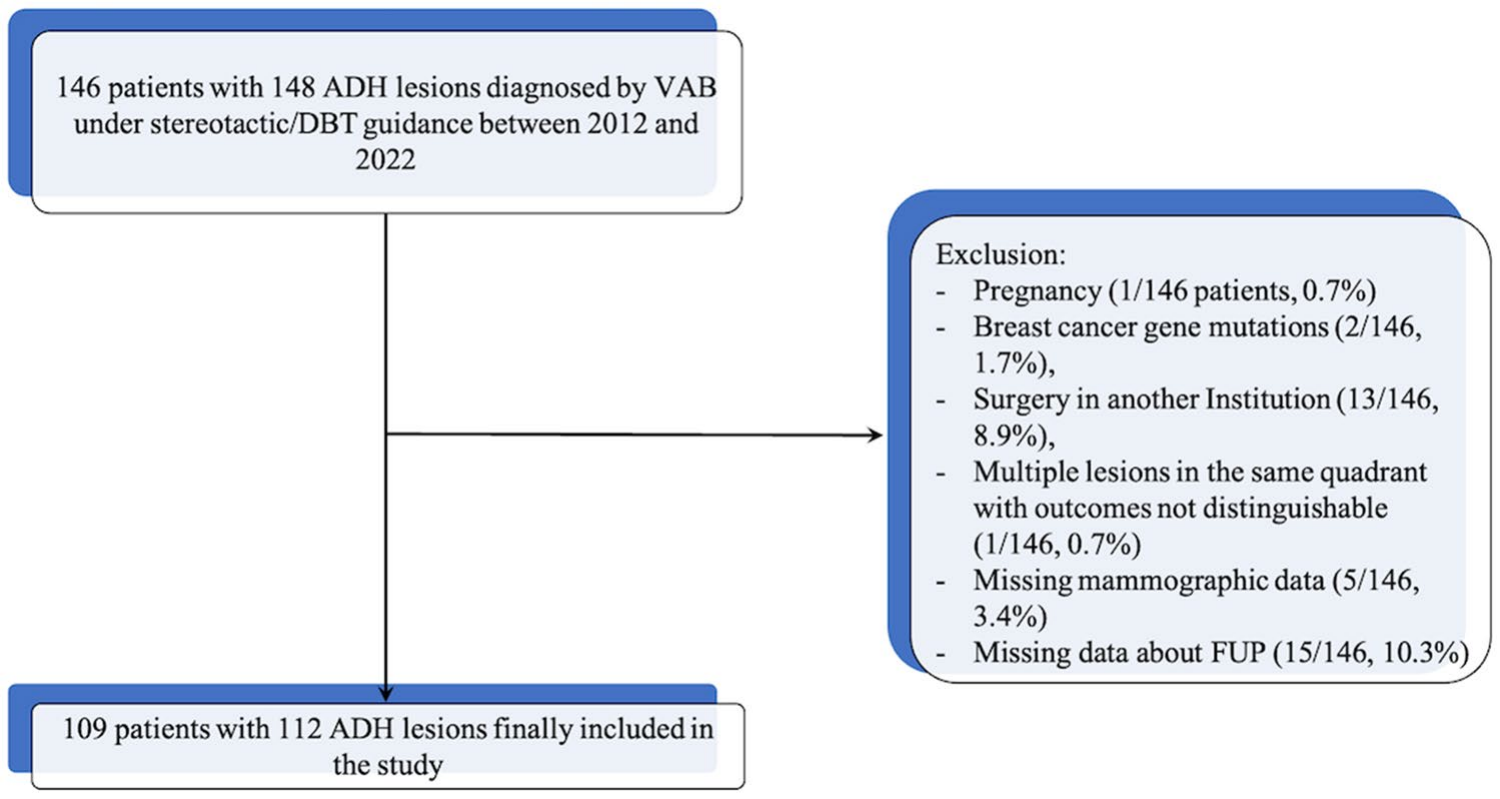
Flow chart diagram of patient selection

Patients with a diagnosis of ADH on VAB under stereotactic/tomosynthesis guidance from 2012 to 2022 were eligible for this study. Inclusion criteria were: availability of diagnostic surgical excision with histopathological examination of the entire lesion or radiologic follow-up (FUP) ≥ 24 months. A total of 146 women with 148 ADH lesions diagnosed by VAB under stereotactic/DBT guidance were initially identified. Pregnant women (1 of 146 patients, 0.7%), women with breast cancer gene mutations (2 of 146 patients, 1.7%), women who underwent surgery in another institution (13 of 146 patients, 8.9%), those with missing mammographic data (5 of 146 patients, 3.4%) or those without data about FUP (15 of 146, 10.3%) and those with multiple lesions in the same quadrant with outcomes not distinguishable (1 of 146 patients, 0.7%) were excluded. Patients with concurrent ipsilateral breast cancer were included when the sites of BC and ADH were separate, with the possibility to identify the surgical histopathological examination of ADH lesion. Finally, 109 patients with 112 ADH lesions were included in the analysis. The data selection process is given in Fig. 1.

### Biopsy procedure

All stereotactic or DBT-guided breast biopsies were performed using 9G (Eviva or Suros ATEC® by Hologic, Marlborough, MA) or 11G (Mammotome® by Devicor Medical Products, Cincinnati, OH, USA) VAB devices, with 6–12 cores obtained from each biopsy site. When more than one target lesion in the same patient was identified, each one of them was biopsied and individually analysed.

### Data collection

Clinical analysed data were abstracted from the electronic medical record and comprised age, premenopausal or postmenopausal status, family history of breast cancer, hormonal therapy, prior malignancy of the breast and concurrent ipsilateral or contralateral breast cancer.

Imaging and histologic features were collected with blinding to the outcome of cancer upgrade. The readings were performed by on-site readers. For each case, the pre-VAB magnified views (if target lesion was microcalcifications) and the pre- and post-biopsy mammograms were reviewed by two breast radiologist for Institution (with > 5 years’ experience in breast imaging) for the following imaging features: breast density, size of target lesion (maximum diameter), lesion type (opacity, microcalcifications, architectural distortion, asymmetry), American College of Radiology Breast Imaging Reporting and Data Systems (BI-RADS) 5th edition assessment category [16], microcalcifications in the retrieved cores (if target lesion was microcalcifications), complete removal of target lesion (checked with mammogram performed immediately after the biopsy) and radiology–pathology correlation. Mammographic breast density, Breast Imaging Reporting and Data System (BIRADS) category of suspicion and radiology–pathology correlation were assigned by the two breast radiologists in consensus.

Original slides from the VAB were reviewed by a pathologist of each Institution (all with more than 20 years’ experience and with special interest in breast pathology), for the following histologic features: number of foci of ADH, ADH pattern (solid, cribriform, micropapillary, other), ADH only in cores with microcalcifications (if target lesion was microcalcifications) and presence of any other B3 lesion.

### Statistical analysis

The VAB examination results were compared with the surgical pathological results or with the imaging follow-up evolution to assess upgrade. The finding of cancer at surgical excision or during FUP is termed “upgrade”.

A backward stepwise linear regression was used to identify possible predictors of the outcome (ADH upgrade). At each step, variables were eliminated based on p values. The discriminatory power of the model was calculated through the ROC AUC. The Hosmer–Lemeshow test was used to assess model calibration.

To adapt the model to clinical work, a procedure similar to the development of the Framingham Risk Score [17] was applied. The points system was developed based on the risk factors of the multivariate model. The risk factors were organized into categories; if a risk factor is continuous (e.g. age), contiguous classes were set up. Reference values for each category were determined. A base category for each risk factor was chosen and the base category is the category assigned 0 points in the scoring system while categories reflecting worse (higher risk of upgrade) states of the risk factor were assigned positive points and categories reflecting better (lower risk of upgrade) states were assigned negative points. How far each category is from the base category was computed first in terms of regression units and then in points dividing the result by a constant (B, arbitrarily chosen). Finally, the risks (probability of upgrade) associated with each point total were determined [17].

Statistical analyses were performed using SPSS version 12.0 software (SPSS, Chicago, IL).

## Results

### Study population

One hundred and nine patients with 112 ADH lesions were included in the analysis. The mean age at diagnosis was 54 ± 9 (SD) years (range 39–83 years), and the mean lesion size was 19.6 ± 17.8 mm (SD). Figure 1 shows the flow chart of the study.

Of those 112 lesions, 91 (91/112, 81.3%) underwent surgical excision and 20 were diagnosed with a malignancy. Final pathology demonstrated 5 invasive cancers and 15 DCIS only. Of the five invasive cancers, two were tubular carcinomas and three were grade 1 invasive ductal carcinomas (one microinvasive). The remaining 21 ADH lesions (21/112, 18.7%) underwent surveillance by annual follow-up with mammogram and ultrasound (mean follow-up time 48 months), due to patient’s preference or patient’s comorbidities. Further sampling with VAB was performed in 1 cases with confirmation of ADH diagnosis. During follow-up, changes in mammographic findings leading to surgical excision were found in only one patient and pathological analysis of surgical excision revealed a malignant lesion (intermediate-grade invasive ductal carcinoma).

The overall upgrade rate was 18.7% (21/112), with a 5.3% (6/112) upgrade rate to invasive cancer and 13.4% (15/112) upgrade rate to DCIS only. Upgrade rates for surgically excised lesions and lesions treated with follow-up were 22.0% (20/91) and 4.8% (1/21), respectively.

### Predictors of upgrade

**Fig. 2:**
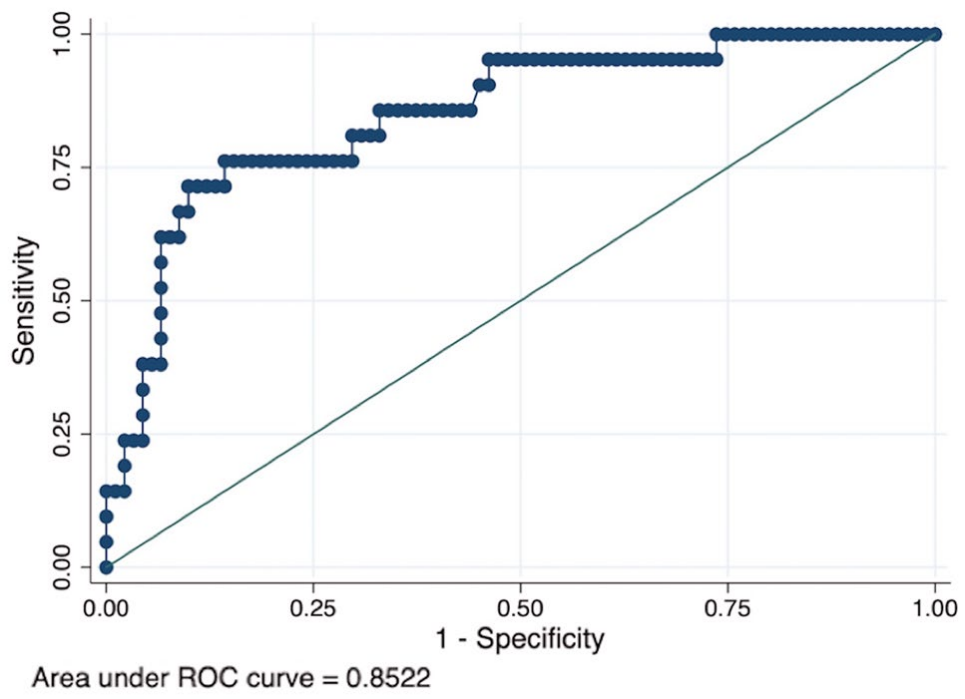
The receiver operating characteristic (ROC) curve for the scoring system. Area under the ROC curve = 0.85 (95% confidence interval 0.76–0.94)

Table 1 summarizes patient characteristics (clinical, radiologic and histopathologic factors). Backward stepwise regression analysis identified age, menopausal status, concurrent breast cancer, BIRADS classification and the number of foci of ADH as risk factors for subsequent ADH upgrade (Table 2). Our model showed an AUC = 0.85 (95% CI 0.76–0.94) (Fig. 2).

**Table 1:** Patient characteristics and cancer upgrade

	N (%) of total (n = 112)	N (%) with upgrade (***n*** = 21)
Clinical features		
Age	54 ± 8.79	59 ± 9.93
Menopausal status		
Premenopausal	55 (49.1)	10 (18.2)
Postmenopausal	57 (50.9)	11 (19.3)
Family history of breast cancer		
Yes	33 (29.5)	6 (18.2)
No	79 (70.5)	15 (19.0)
Hormone therapy		
Yes	33 (29.5)	6 (18.2)
No	79 (70.5)	15 (19.0)
History of breast cancer		
Yes	4 (3.6)	1 (25.0)
No	108 (96.4)	20 (18.5)
Concurrent breast cancer (ipsi or controlateral)		
Yes	4 (3.6)	3 (75.0)
No	108 (96.4)	18 (16.7)
Radiologic features		
Breast density		
ACR category a–b	48 (42.9)	11 (22.9)
ACR category c–d	64 (57.1)	10 (15.6)
Type of mammographic lesion		
Calcifications	98 (87.5)	17 (17.3)
Others (mass, architectural distortion, asymmetry)	14 (12.5)	4 (28.6)
Lesion size (maximum diameter)		
< 2 cm	75 (67.0)	9 (12.0)
> 2 cm	37 (33.0)	12 (32.4)
BIRADS classification		
3–4A	42 (37.5)	4 (9.5)
4B–4C–5	70 (62.5)	17 (24.3)
Guidance		
Stereotactic		
Tomosynthesis		
Biopsy needle gauge		
11G	57 (50.9)	7 (12.3)
9G	55 (49.1)	14 (25.4)
Post-biopsy residual lesion		
Yes	66 (58.9)	15 (22.7)
No	46 (41.1)	6 (13.0)
Histopathological variables		
Number of foci ADH		
Single	65 (58.0)	4 (6.1)
Multiple	47 (42.0)	17 (36.2)
Pattern of ADH		
Micropapillary	9 (8.0)	2 (22.2)
Other	57 (50.9)	15 (26.3)
Missing	46 (41.1)	4 (8.7)
ADH only in cores with microcalcifications		
Yes	51 (45.5)	11 (52.4)
No	61 (54.5)	10 (47.6)
Presence of any other high-risk lesion		
Yes	76 (67.9)	14 (18.4)
No	36 (32.1)	7 (19.4)
Radio-pathological discordance		
Yes	9 (8.0)	3 (33.3)
No	103 (92.0)	18 (17.5)

Numeric data are presented as mean ± standard deviation. Nonnumeric data are presented as numbers of lesions with percentages in parentheses

ACR, American College of Radiology; ADH, atypical ductal hyperplasia; BIRADS, Breast Imaging Reporting and Data System

**Table 2:** Multivariate logistic regression model

Variables	OR	95% CI	*P* value
Age	2.54	1.05–6.16	0.038
Menopausal status			
Premenopausal	Reference		
Postmenopausal	0.18	0.033–0.99	0.049
Concurrent breast cancer (ipsi or controlateral)			
Yes	31.82	1.20–843.81	0.039
No	Reference		
BIRADS classification			
3–4A	Reference		
4B–4C–5	5.60	1.06–29.52	0.042
Number of foci ADH			
Single	Reference		
Multiple	7.48	2.11–26.60	0.002

ADH, atypical ductal hyperplasia; BIRADS, Breast Imaging Reporting and Data System; CI, confidence interval; OR, odds ratio

### Scoring system

**Fig. 3:**
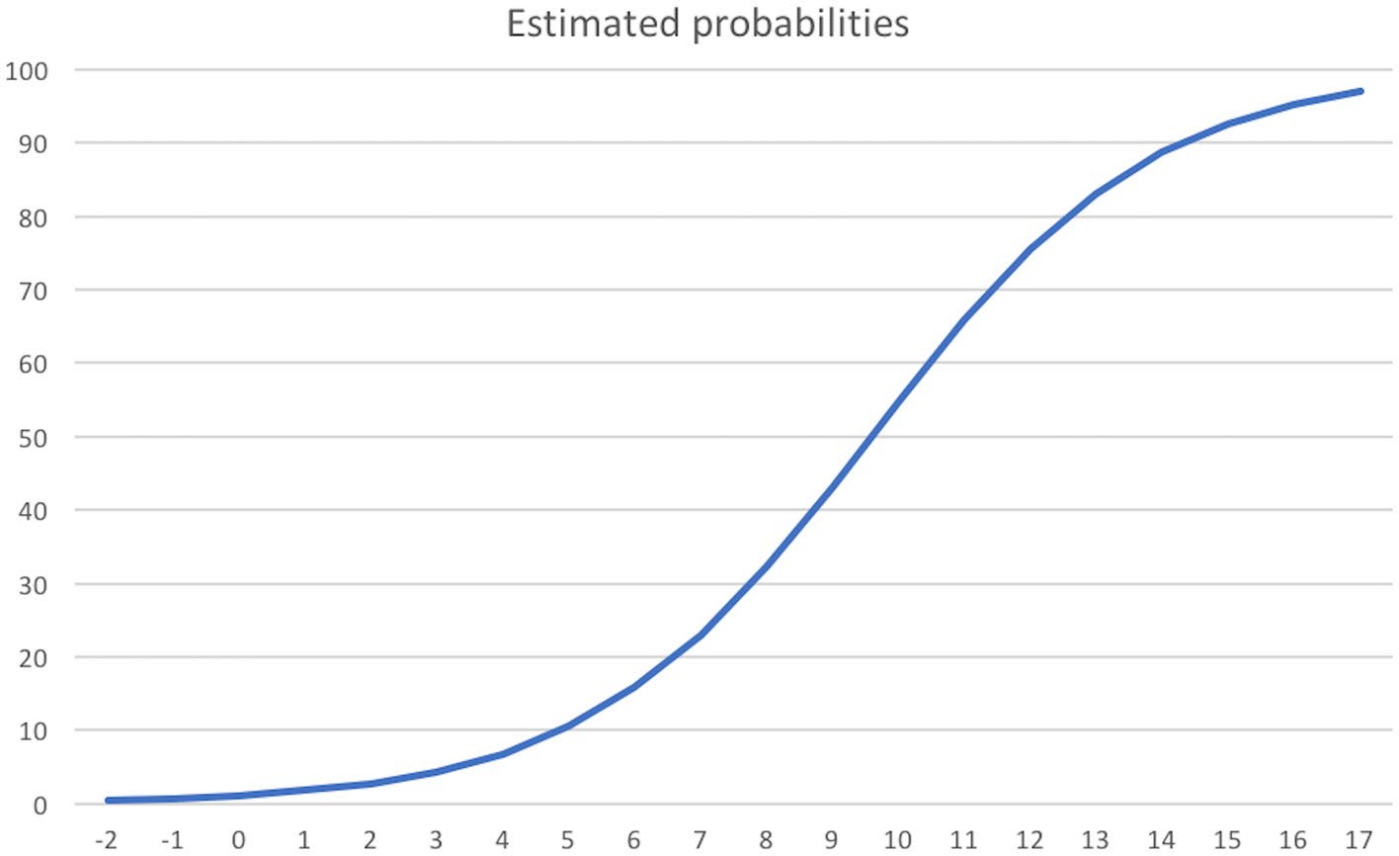
Line graph showing the relationship between the point total (x-axis) and the risk of upgrade (y-axis)

**Fig. 4:**
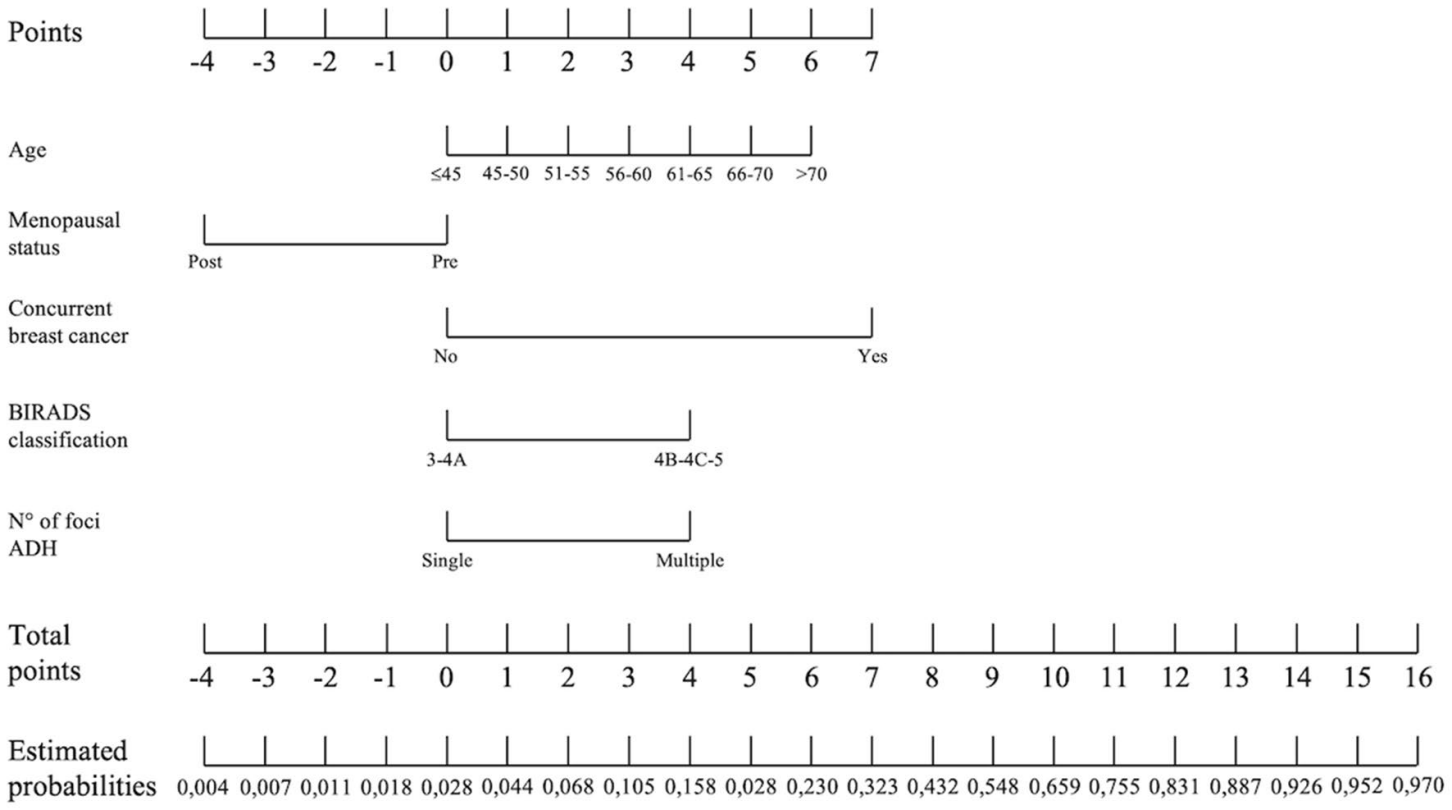
Nomogram for predicting the risk of ADH upgrade

**Fig. 5:**
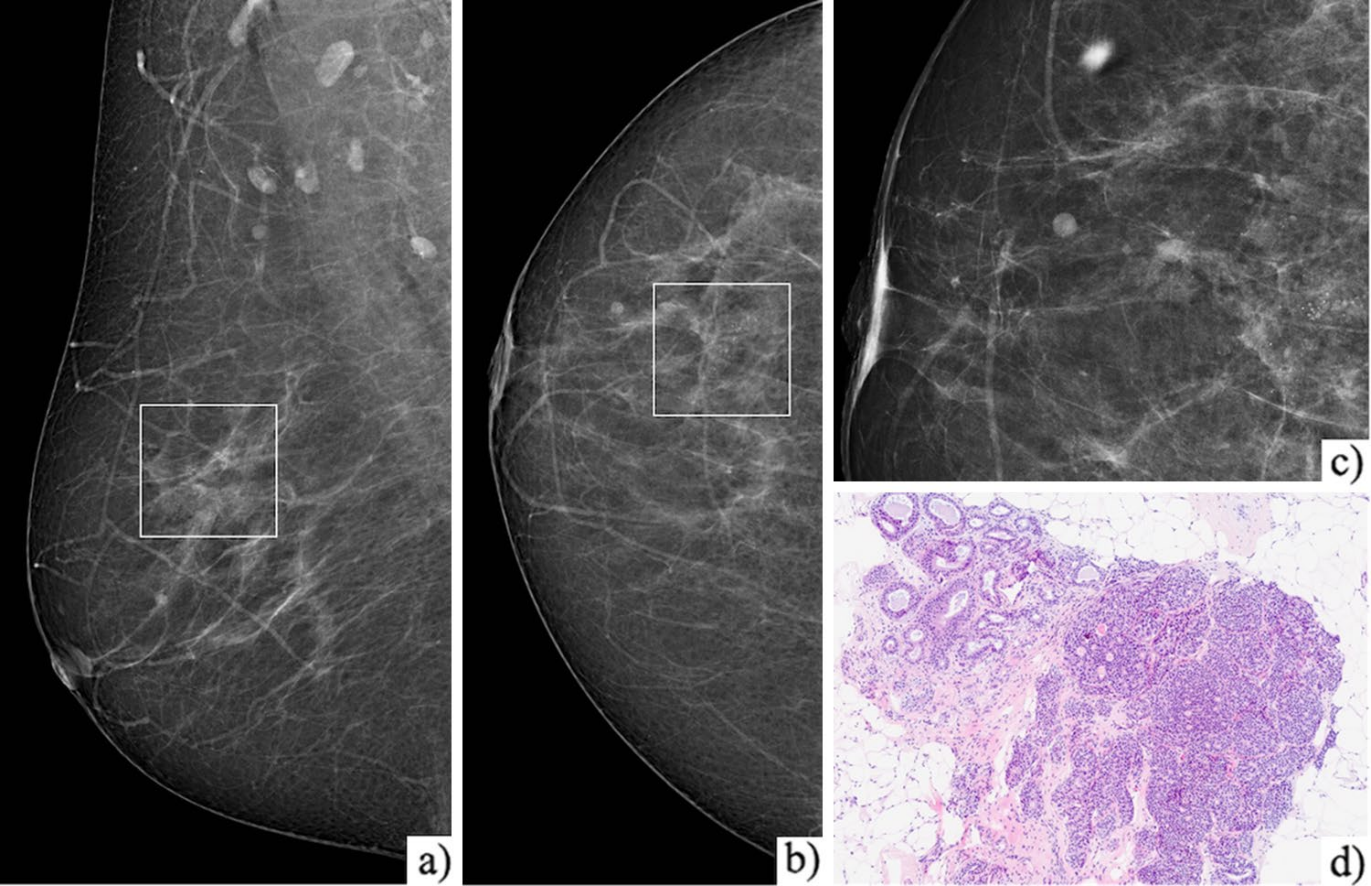
An example of the use of the model. Mediolateral oblique **a**, craniocaudal **b** and magnification **c** views of the right breast in a 46 years woman (1 point) in post-menopausal status (-4 points), without concurrent breast cancer (0 points), with microcalcifications classified as BIRADS 4B (4 points), with flat epithelial atypia with a single focus of ADH at VAB histopathological analysis (0 points) **d**. Using the developed points system, a final score of 1 was assigned with a corresponding risk < 2%. This lesion underwent surgical excision with only benign findings

Based on the selected independent risk factors, we developed a points system to predict the probability of upgrade. The reference values, beta coefficients and points of each category of the significant factors in the multivariate model are shown in Table 3. Table 4 shows the estimated probabilities of upgrade associated with each point total, with increasing risk when the score increases (Fig. 3). Based on these results, a nomogram was created (Fig. 4). When the total score is ≤ 1, the risk of upgrade is less than 2% (Fig. 5).

**Table 3:** Reference values, beta coefficients and points of each category of the significant factors in the multivariate model

Risk factor	Categories	Reference value (Wi)	Beta	Beta (Wi-Wref)	B	Beta (Wi-Wref)/B
Age	< = 45	42,5	0,093424	0	0,467120	0
	> 45–50	47,5	0,093424	0,46712	0,467120	1
	> 50–55	52,5	0,093424	0,93424	0,467120	2
	> 55–60	57,5	0,093424	1,40136	0,467120	3
	> 60–65	62,5	0,093424	1,86848	0,467120	4
	> 65–70	67,5	0,093424	2,3356	0,467120	5
	> 70	72,5	0,093424	2,80272	0,467120	6
Menopausal status	Pre	0	− 1,714877	0	0,467120	0
	Post	0	− 1,714877	− 1,714877	0,467120	− 4
Concurrent breast cancer	No	0	3,460178	0	0,467120	0
	Yes	0	3,460178	3,460178	0,467120	7
BIRADS classification	3–4A	0	1,723341	0	0,467120	0
	4B–4C–5	0	1,723341	1,723341	0,467120	4
N° of foci ADH	Single	0	2,012696	0	0,467120	0
	Multiple	0	2,012696	2,012696	0,467120	4

ADH, atypical ductal hyperplasia; BIRADS, Breast Imaging Reporting and Data System; CI, confidence interval; OR, odds ratio

**Table 4:** Estimated probabilities of upgrade of each point total of the scoring system

Points	Estimated probabilities
− 2	0004
− 1	0007
0	0011
1	0018
2	0028
3	0044
4	0068
5	0105
6	0158
7	0230
8	0323
9	0432
10	0548
11	0659
12	0755
13	0831
14	0887
15	0926
16	0952
17	0970

The estimated probabilities are expressed as decimal

## Discussion

The present study is the first to develop a scoring system to predict the probability of upgrade in patients diagnosed with ADH at stereotactic/DBT-guided VAB. ADH is still a challenge for breast specialists: although most ADH is benign, surgical excision is still recommended because in the impossibility of identifying a subgroup of these lesions with a sufficiently low upgrade rate to obviate surgery [11, 15]. This leads to a huge number of unnecessary surgical excisions that represent an overtreatment, especially in the era of de-escalation where even for DCIS there is growing evidence that monitoring select cases is a safe alternative to standard surgical therapy [18, 19].

We selected only ADH diagnosed with stereotactic/DBT-guided VAB to reduce heterogeneity of data. Moreover, previous studies and a recent meta-analysis demonstrated that upgrade rate is significantly lower (about half) in this type of biopsy if compared to US-guided or MRI-guided biopsies [12–15], probably related to lesion type (prevalence of microcalcifications), larger core specimens and using the vacuum-assisted device, so a part of these lesions could be potentially suitable for follow-up instead of surgical excision, if correctly identified. We also decided to exclude mutations carrier patients, due to the high malignancy association of B3 lesions in this category of patients, which suggests that these lesions must always be surgically excised in high-risk women [20].

In our study, the underestimation rate was 18.7%, in line with previously published results [9, 10, 15, 21] and, even when only upgrade rate to invasive cancers is considered, a 5.3% of underestimation was found, indicating a too high percentage to safely avoid surgery. The present study identified higher age, concurrent breast cancer, 4b/4c/5 BIRADS classification and multiple foci of ADH as independent predictors of upgrade, while post-menopausal status was a negative predictor. We found that younger and pre-menopausal women are at higher risk of upgrade, as previously found by several studies [22–24]. Also, BIRADS classification of the lesion was confirmed to be a factor associated with upgrade [11]. The presence of an ipsilateral or contralateral BC resulted significantly associated with the risk of upgrade, justifying the simultaneous excision of both the breast cancer and the site of ADH (with one large lumpectomy or two separate lumpectomies) to accomplish the goal of removing the known cancer and ruling out additional malignancy at the site of ADH. Our results also confirmed that multiple foci of ADH on biopsy are associated with more frequent upgrade, as previously reported in several previous studies, using a cut-off of either < 2 foci [25, 26] or < 3 foci [27, 28]. We did not found a statistically significant difference in the upgrade rate between stereotactic and DBT guidance: most of ADH lesions (87.5% in our cohort) present as microcalcifications that can be correctly identified and biopsied under stereotactic guidance, without a significant improvement when DBT guidance is used (as we would expect for architectural distortions, better depicted with DBT). The complete removal of the target lesion did not enter our model. This factor was first considered as a safe condition to justify follow-up, but was subsequently demonstrated that the upgrade rate (also in cases with complete removal of target lesion) was still too high and the recommendation for follow-up in these cases was removed from the second edition of the consensus conference on B3 lesions [5]. The dimension of the lesion was not identified as a predictor of upgrade, too, while most of the previously published studies found a significant association between residual lesion and upgrade [11, 25, 26] and also the univariate analysis of our data demonstrated an association between diameter of the lesion and upgrade (*p* = 0.012, data not shown). However, since the purpose of our study is to identify possible predictors of the outcome (ADH upgrade) to build a scoring system, we used a backward stepwise linear regression, which builds a regression model from a set of candidate predictor variables by removing predictors based on p values, in a stepwise manner, using an automated method. Stepwise regression did not confirm the results of univariate analysis, probably because the diameter of the lesion has a less significant impact on upgrade if compared to the other predictors that entered the model. About ADH pattern, contrasting results have been published [25, 27] and our results did not find that micropapillary pattern significantly increased the risk of malignancy. Previous studies reported that the upgrade rate is significantly reduced when ADH is found only in specimens showing microcalcifications [11], while our data did not confirm this association. Finally, our data confirmed previously published results which showed that there is no association between ADH upgrade and the presence of other B3 lesions [12, 29–32]; it seems that, when ADH is present, its own risk of upgrade overwhelms the risk associated with other B3 lesions such as FEA, LN, papilloma or RS.

Our model showed an AUC = 0.85 (95% CI 0.76–0.94), indicating a good discriminatory power. A scoring systems was then developed as a statistical tool to predict the probability of upgrade and assist clinicians in decision-making. Ko et al. [33] previously proposed a scoring system for ADH diagnosed at ultrasound-guided CNB based on clinical, imaging and pathologic features, but they tested this score in only 34 patients and a subsequent validation study [34] demonstrated the low reproducibility of this score. The present scoring system demonstrated a high diagnostic performance to identify women at low risk (< 2%) of malignant upgrade when the total score is ≤ 1, thus this cut-off value can be used to define a subset of “probably benign” lesions, corresponding to a BIRADS category 3. These lesions could be safely sent to follow-up, reducing over-treatment and consequently morbidity and economic burden. Even if novel approaches, such as artificial intelligence [35, 36] or molecular markers [37, 38], seem to be promising ways forward, our tool, based on clinical, radiological and histopathological data, easy to retrieve in any reality, can be a useful tool in daily practice.

Limitations of the present study include its retrospective design and lack of a dataset to validate the performance of our model so we are working to validate our work prospectively on a larger, independent cohort. Moreover, our model is built only on ADH diagnosed with VAB under stereotactic/DBT guidance so it can be useful for decision-making only for a subset of ADH diagnosis. Lastly, we included in the study also women who did not undergo surgery (21/112, 18.7%) so, even if the median follow-up time is relatively long (48 months), it could be possible that indolent low-grade in situ cancer in patients who were managed conservatively may have not become evident in the imaging follow-up period.

In conclusion, our scoring system, based on clinical, radiologic and histopathologic parameters, seemed a promising easy-to-use decision support tool for management of ADH, decreasing unnecessary surgeries, reducing patients’ overtreatment and healthcare costs. Further work is needed to validate our model on independent datasets.
